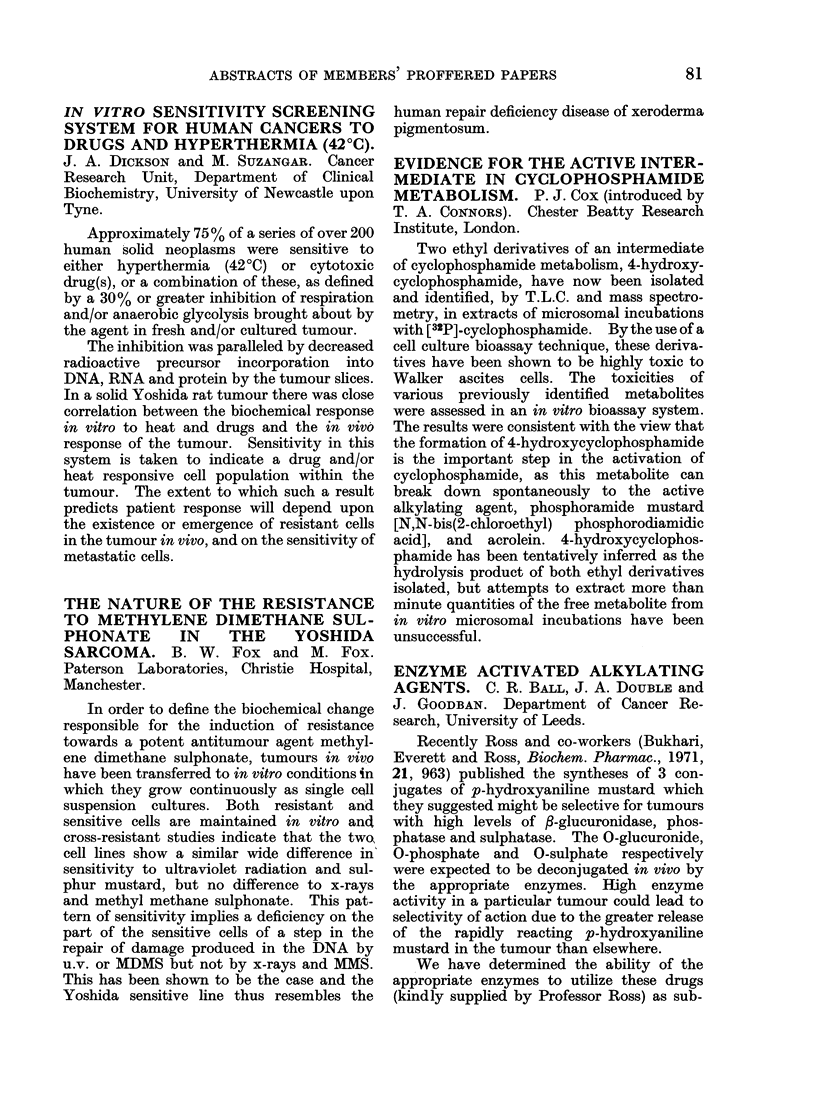# The nature of the resistance to methylene dimethane sulphonate in the Yoshida sarcoma.

**DOI:** 10.1038/bjc.1973.90

**Published:** 1973-07

**Authors:** B. W. Fox, M. Fox


### THE NATURE OF THE RESISTANCE TO METHYLENE DIMETHANE SULPHONATE IN THE YOSHIDA SARCOMA.

B. W. FOX and M. FOX. Paterson Laboratories, Christie Hospital, Manchester.

In order to define the biochemical change responsible for the induction of resistance towards a potent antitumour agent methylene dimethane sulphonate, tumours *in vivo* have been transferred to *in vitro* conditions in which they grow continuously as single cell suspension cultures. Both resistant and sensitive cells are maintained *in vitro* and cross-resistant studies indicate that the two cell lines show a similar wide difference in sensitivity to ultraviolet radiation and sulphur mustard, but no difference to x-rays and methyl methane sulphonate. This pattern of sensitivity implies a deficiency on the part of the sensitive cells of a step in the repair of damage produced in the DNA by u.v. or MDMS but not by x-rays and MMS. This has been shown to be the case and the Yoshida sensitive line thus resembles the human repair deficiency disease of xeroderma pigmentosum.